# Transcatheter Mitral Valve Replacement in High-Surgical Risk Patients: A Single-Center Experience and Outcome

**DOI:** 10.1155/2022/6587036

**Published:** 2022-06-22

**Authors:** Fatma A. Taha, Hesham Naeim, Fareed Alnozha, Osama Amoudi, Reda Abuelatta

**Affiliations:** ^1^Adult Cardiology Department, Madinah Cardiac Center, Madinah, Saudi Arabia; ^2^Cardiology Department, Faculty of Medicine, Tanta University, Tanta, Egypt

## Abstract

**Background:**

Re-operative mitral valve (MV) replacement is a high-risk procedure, therefore, transcatheter MV replacement (TMVR) is a promising therapeutic option.

**Aim:**

In this study, we aimed to evaluate the feasibility and safety of TMVR in patients with high surgical risk with degenerated mitral bioprostheses (TMViV), failed surgical rings (TMViR), and mitral annular calcification (TMViMAC).

**Methods:**

This is a retrospective cohort study that enrolled patients with high surgical risk who underwent TMVR from February 2017 to September 2020. The TMVR procedure was performed using Edwards SAPIEN-3 valves through the transseptal approach.

**Results:**

Sixty-four patients aged 62.7 ± 16.1 years with an STS score of 9.2 ± 3.7% underwent TMVR [35 (55%) TMViV, 16 (25%) TMViR, and 13 (20%) TMViMAC]. Mitral stenosis was more frequent in TMViV, mitral regurgitation was more frequent in TMViR, and combined mitral stenosis and regurgitation were more frequent in TMViMAC (*P* < 0.05). The MV gradient was 14.3 ± 5.3 mmHg and the MV area was 1.5±0.6 cm^2^. The 29 mm valve was frequently used in TMViV and TMViMAC, while the 23 mm valve was frequently used in TMViR (*P*=0.003^*∗*^). The procedural and fluoroscopy times were 58.7 ± 8.9 and 41.1 ± 8.2 minutes, respectively. Technical success was reported in 62 (98.4%) patients; 1 TMViR patient experienced valve embolization and salvage surgery, and 1 TMViMAC patient experienced slight valve malposition. At 3 months, 2 (3.1%) patients showed valve thrombosis (treated with anticoagulation), and 1 (1.6%) patient developed a paravalvular leak (underwent surgical MV replacement). At 6 months, 3 (4.7%) patients showed valve degeneration (underwent surgical MV replacement). Throughout follow-up, no patient exhibited mortality.

**Conclusions:**

TMVR is a feasible and safe approach in patients with high surgical risk. TMViV and TMViR are reasonable as the first treatment approaches, and TMViMAC seems encouraging.

## 1. Introduction

Mitral valve (MV) disease is the most common valve disease [1, 2], with mitral regurgitation (MR) representing the commonest MV lesion, considering the reduction in rheumatic heart disease and the subsequent mitral stenosis (MS) [1]. Moreover, the growing incidence of ischemic heart disease and the degenerative valvular lesions has increased the ischemic secondary and the degenerative primary MR [3]. However, the conventional MV surgery (repair or replacement) remains the gold standard treatment for patients with severe symptomatic MR [4]; close to half of such patients have potential comorbidities and are not candidates for surgery. In recent years, several transcatheter MV technologies have emerged as alternatives to surgery for the treatment of MR in patients with high surgical risk as the MV clipping technique. However, a percentage of patients are suboptimal candidates for this technology, with a residual moderate-to-severe MR has been reported in about 10% of patients [5, 6].

Also, reoperative MV replacement is a complex and invasive procedure; the technical challenges of re-entering the chest are often combined with the medical comorbidities of the patient. Thus, a growing interest in a transcatheter approach for the management of MV disease in patients with failed MV after previous cardiac surgery has evolved [7, 8]. In such patients with high surgical risk, those denying surgery, and those with unsatisfactory medical therapy, transcatheter mitral valve replacement (TMVR) using transcatheter balloon-expandable aortic heart valves is a promising therapeutic option [7, 8]. TMVR includes transcatheter mitral valve-in-valve (TMViV) replacement for degenerated mitral bioprostheses, transcatheter mitral valve-in-ring (TMViR) implantation for failed surgical rings, and transcatheter mitral valve-in-mitral annular calcification (TMViMAC) insertion for a native valve with severe mitral annular calcification (MAC). TMViV procedures for patients with high surgical risks were approved by the Food and Drug Administration (FDA) in the United States in 2017, while TMViR and TMViMAC procedures remain off-label. Limited data from registries suggest that TMViV and TMViR are feasible with reasonable outcomes in patients with high surgical risks [9–11]. The MITRAL (mitral implantation of transcatheter valves) trial recently demonstrated early outcomes in patients who underwent TMVR with Edwards SAPIEN-XT and SAPIEN-3 (Edwards Lifesciences) transcatheter heart valves (THVs) for bioprosthetic valves, failing surgical rings, and severe mitral annular calcification [12, 13].

In this study, we aimed to evaluate the feasibility and safety of TMVR in patients with high surgical risks with degenerated mitral bioprostheses, failed surgical rings, and severe mitral annular calcification of native MV, and to compare the 3 groups as regards the preprocedure characteristics, procedural measures, and their 6-month postprocedural outcomes.

## 2. Methods

### 2.1. Study Design

This is a single-center retrospective cohort study that enrolled all patients with high surgical risk who underwent TMVR, TMViV for degenerated mitral bioprostheses, TMViR for failed surgical rings, and TMViMAC for native MV with severe MAC from February 2017 to September 2020. There were no patients excluded from TMVR consideration secondary to the valve or ring type. The 3 groups' (TMViV, TMViR, and TMViMAC) data were collected, analyzed, and compared. This study complied with the ethical guidelines of the Declaration of Helsinki (as revised in 2013) and was approved by the institutional review board committee. Informed written consent was obtained from all patients before the procedure and the patient's consent to participate in the study was waived because of the retrospective nature of the study.

### 2.2. Retrospective Quality Review

Patients' medical records were reviewed with the consideration of the following: (1) demographic characteristics of previous cardiac history and prior surgical prostheses; (2) clinical presentations including New York Heart Association Functional Classification (NYHA-FC); (3) transthoracic echocardiography (TTE) data of the MV pathology and lesions; (4) cardiac catheterization screening for coronary artery disease; (5) cardiac computed tomography (CT) assessment of the true inner diameter of the bioprostheses/ring/native valve and screening of the neo-left ventricular outflow tract (LVOT) obstruction risk. All the studied patients were discussed by the institute's advanced intervention heart team and were considered at high or prohibitive Society of Thoracic Surgeons (STS) risk for MV surgery. Acute infective endocarditis was excluded in all patients.

### 2.3. Echocardiography

All patients underwent a thorough TTE assessment of the bioprosthesis/ring/native valve morphology and hemodynamics before, during, and immediately after valve implantation using PHILIPS-iE33 and PHILIPS-EPIQ-CVx echocardiography (USA). Preprocedural transesophageal echocardiography (TEE) was conducted to confirm the findings of the TTE and to exclude the left atrial appendage thrombus. Intraprocedural TEE and three-dimensional TEE (3D-TEE) guided the septal puncture, the valve positioning, and the whole procedure. Also, it was essential for the assessment of transmitral gradients, the presence of central or paravalvular leaks, the motion of valvular leaflets after TMVR, and any prosthesis encroachment on the LVOT. Routine TTE was performed on the first day after the procedure, before hospital discharge, and at 3- and 6-month follow-ups.

### 2.4. Transcatheter Mitral Valve Replacement (TMVR) Technique

#### 2.4.1. Valve Sizing

An Edwards SAPIEN-3 prosthesis (Edwards Lifesciences, Irvine, CA, USA) was used in all patients. The size of the THV was selected using the manufacturers' internal dimensions, the TEE measurements, and the CT assessments. In patients who underwent TMViV and TMViR implantation, the Valve in Valve (Mitral) app developed by Dr. Vinayak Bapat, MD (UBQO Limited), was used for sizing of the THV [14]. In patients who underwent TMViMAC implantation, a 3D-CT evaluation was performed to determine the exact dimensions of the calcified annulus and for confirmation of >50% calcification of the MV circumference. 10% oversizing of the implanted THV than the individual calcified native mitral annulus was considered optimal for better anchoring.

#### 2.4.2. Access

Transfemoral access with an antegrade transseptal puncture was performed in all patients, under TEE and fluoroscopic guidance. A 0-1 Brockenbrough needle (BRK™, Abbott Vascular, IL, USA) was rotated clockwise inside an 8.5F-SL sheath (St. Jude Medical) to achieve a posterior-superior septal puncture. After securing the transseptal puncture by visualizing both the needle and the bubbles in the left atrium (LA), 100 IU/kg of unfractionated heparin was administered with booster doses to maintain an activated clotting time of >250 seconds. The femoral venous access was closed by a figure of 8 stitches.

#### 2.4.3. Procedure Details

After the transseptal puncture, an 8.5F-Agilis™ NxT steerable sheath (Abbott Vascular, IL, USA) was advanced to the LA. The Agilis™ catheter was flexed and directed toward the MV bioprosthesis, the annuloplasty ring, or the native MV and was used to navigate a 0.035-inch curved Terumo guidewire (Somerset, NJ, USA) from the LA aspect through the MV to the left ventricle (LV), then to the aorta. Afterward, the guidewire was exchanged for an extra-stiff Confida™ Brecker guidewire (Medtronic Inc, Minneapolis, Minnesota, USA) that was secured into the LV apex over a 5F multipurpose, Judkins right, or pigtail catheter. Mostly, another super-stiff wire with its end manually bent as a J-curve (e.g., Amplatz™ super-stiff wire (Abbott Vascular, IL, USA)) was introduced to the LV apex for reaccessing if the first wire lost its position. Then, balloon dilatation of the interatrial septum was performed using a 12 or 14 mm balloon to facilitate the passage of the THV across the septum.

Thereafter, a 16F Edwards sheath was then secured in the femoral vein over the stiff wire. In some patients, pre-TMVR balloon dilatation of the stenosed bioprosthesis/ring/native valve was performed. Afterward, transseptal insertion of the Edwards Commander delivery system (Edwards life sciences, Irvin California, USA) with the mounted Edwards SAPIEN-3 THV (mounted in the opposite direction for the transfemoral transcatheter aortic valve implantation (TAVI)) was inserted into the MV. The aim was to place the prosthesis with its outer skirt exactly into the plane of the bioprosthesis/ring/calcified annulus. This was achieved by a slight protrusion of approximately 10–20% of the prosthesis into the LA. An additional post-TMVR distal valve flaring for about 10% more than the annulus was performed using the same Edwards system balloon. No ventricular pacing was used at any stage of the TMVR procedure.

#### 2.4.4. Procedural Safety and Quality Measures

Complete technical success was defined by the ability of the SAPIEN-3 valve to be deployed in an accurate position through the planned access, without the need for emergency surgery/reintervention and without procedural mortality [15]. Clinical success was defined as the in-hospital post-TMVR improvement of the NYHA-FC by at least one grade. Procedural complications were assessed according to the Mitral Valve Academic Research Consortium (MVARC) criteria [15], and procedural mortality was defined as any occurring death within 24 hours after the procedure. All postoperative complications were recorded, including the need for the operative valvular reintervention.

### 2.5. Antithrombotic Regimen

All patients received either a loading dose of 300 mg of clopidogrel or 180 mg of ticagrelor before the procedure. Post-TMVR: (1) In patients without an indication for long-term oral anticoagulation and dual antiplatelet therapy (DAPT), 100 mg aspirin and 75 mg clopidogrel or 180 mg of ticagrelor were given for one year. (2) In patients with an indication for permanent oral anticoagulation (warfarin or apixaban), 75 mg clopidogrel or 180 mg of ticagrelor was added to the anticoagulant for 3–6 months.

### 2.6. Statistical Analysis

Statistical analysis was performed using the SPSS statistical package (Version 25; SPSS Inc., Chicago, IL, USA). Continuous variables were expressed as mean ± standard deviation and categorical variables were expressed as numbers and percentages. The analysis of variance (ANOVA) test was used to compare the distributions of the 3 groups for the continuous variables and the chi-square test was used for the categorical variables. The paired sample *t*-test and the chi-square test were performed to compare the preprocedural and postprocedural categorical variables and continuous variables, respectively. A *P* value of <0.05 was considered statistically significant at a confidence interval of 95%.

## 3. Results

### 3.1. Baseline Demographic, Clinical, and Echocardiographic Characteristics

A total of 64 patients with high surgical risk underwent clinically indicated TMVR, 35 (55%) patients underwent TMViV, 16 (25%) patients underwent TMViR, and 13 (20%) patients underwent TMViMAC. The mean age was 62.7 ± 16.1 years with significantly older patients in the TMViMAC group (*P*=0.024). Thirty-five (54.7%) patients were females, one of them presented with severe heart failure (HF) during her 5th month of pregnancy and required salvage TMViV intervention. TMViR patients were more likely to require home oxygen (*P*=0.026), while TMViMAC patients were more likely to have chronic kidney disease/renal transplant (*P* < 0.001), prior stroke (*P*=0.014), and chronic anemia (*P* < 0.001). TMViR patients were more likely to have a prior myocardial infarction (*P* < 0.001) and prior coronary artery bypass graft (*P* < 0.001), while TMViMAC patients were more likely to have prior primary coronary intervention (PCI) (*P*=0.006), prior aortic valve replacement (*P*=0.047), and porcelain aorta (*P*=0.002). In the entire cohort, the mean surgical STS score for morbidity and mortality was 9.2 ± 3.7% without a significant difference between the groups (*P*=0.442). Forty-two (65.63%) patients had at least one admission for HF in the last 12 months, and all patients had NYHA-FC III or IV without a significant difference between the groups (*P*=0.939).

Mitral stenosis was more frequently found in TMViV (*P*=0.043), mitral regurgitation was more frequent in TMViR (*P* < 0.001), and combined mitral stenosis and regurgitation were more frequent in TMViMAC (*P*=0.003). In the entire cohort, the mean MV pressure gradient (Pg) was 14.3 ± 5.3 mmHg, and the MV area was 1.5 ± 0.6 cm^2^ with a significantly smaller area in TMViV patients (*P* < 0.001). MR was valvular in origin in 41 (64.1%) patients and paravalvular in 3 (4.7%) TMViR patients (*P*=0.022). The left ventricular ejection fraction (LVEF) was significantly lower in TMViR patients and LVOT-Pg was significantly higher in TMViMAC patients (*P* < 0.001). Baseline demographic, clinical, and echocardiographic characteristics of the studied patients are summarized in Table 1.

### 3.2. Preprocedural, Procedural, and Postprocedural Safety and Quality Measures

**Figure 1:**
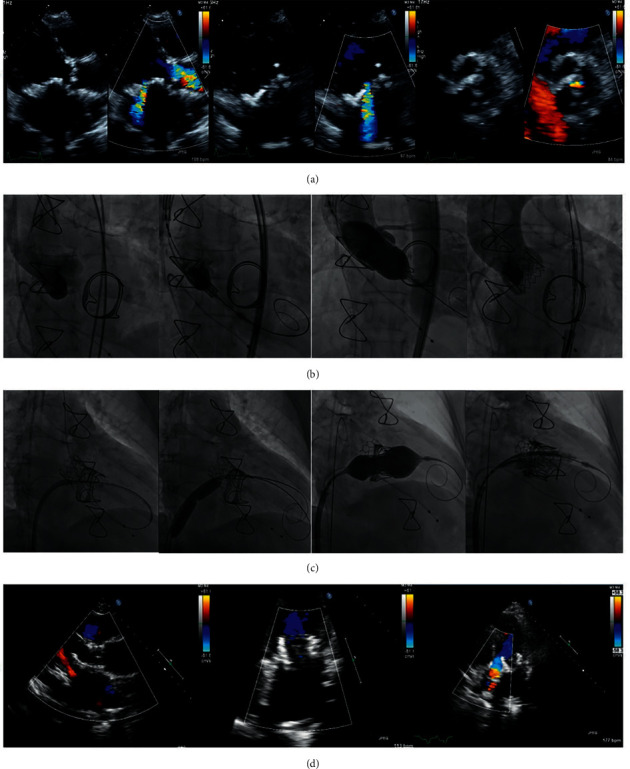
Transcatheter mitral valve in a degenerated Carpentier–Edwards (Edwards Lifesciences) valve (TMViV) with a concomitant transcatheter aortic valve implantation (TAVI). A 64-year-old, diabetic, hypertensive female patient, with COPD and renal transplant, had a degenerated bioprosthetic 29 mm Carpentier–Edwards MV and a degenerated calcific AV with an associated AF. (a) TTE shows a degenerated bioprosthetic MV (severe MS and severe MR) with a calcific degenerated AV (severe AS and mild AR). (b) Fluoroscopy shows a transaortic TAVI of an Edwards SAPIEN-3 23 mm valve during rapid pacing. (c) Fluoroscopy shows an 8.5F-agilis™ sheath including a 5F-MP catheter over a 0.035-inch curved Terumo guidewire to cross the degenerated bioprosthetic MV to the LV, and then to the aorta. Balloon dilatation of the transseptal puncture using a 14 mm balloon over a 0.035-inch/260 extra-stiff Confida™ guidewire. Transseptal TMViV of an Edwards SAPIEN-3 29 mm valve. (d) TTE: both SAPIEN-3 valves are in mitral and aortic positions with normal flow across both valves. AF: atrial fibrillation, AR: aortic regurgitation, AS: aortic stenosis, AV: aortic valve, AVA: aortic valve area, COPD: chronic obstructive pulmonary disease, LV: left ventricle, MR: mitral regurgitation, MS: mitral stenosis, MV: mitral valve, MVA: mitral valve area, Pg: pressure gradient, TAVI: transcatheter aortic valve implantation, TMViV: transcatheter mitral valve-in-valve, and TTE: transthoracic echocardiography.

**Figure 2:**
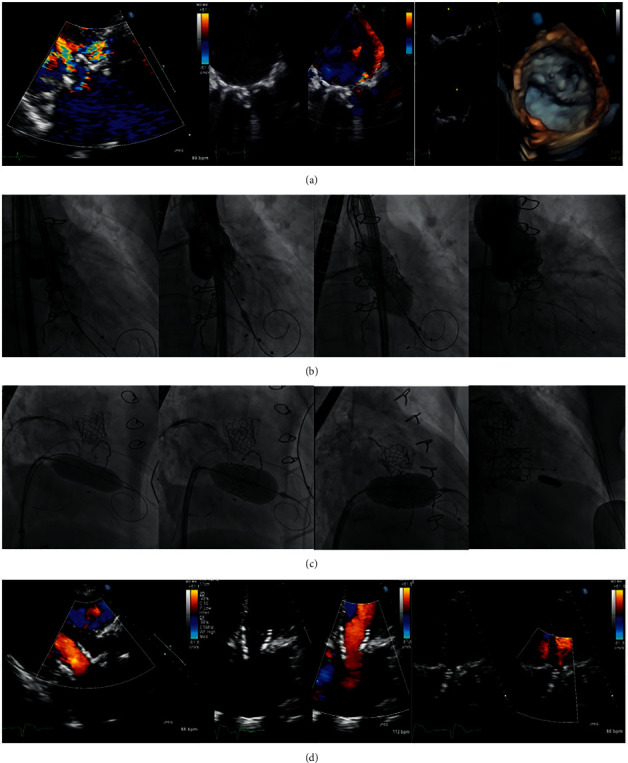
Transcatheter mitral valve in a degenerated Mosaic® ultra™ (Medtronic) valve (TMViV) with a concomitant transcatheter aortic valve implantation (TAVI). A 51-year-old male patient had a degenerated 23-mm Mosaic® ULTRA™ bioprosthetic MV (Medtronic) and a degenerated calcific AV with an associated AF. (a) TTE, TEE, and 3D-TEE show a degenerated bioprosthetic MV (severe MS and severe MR) and a calcific degenerated AV (severe AS and severe AR). (b) Fluoroscopy shows a transaortic TAVI of an Edward SAPIEN-3 26 mm valve during rapid pacing. (c) Fluoroscopy shows pre-TMViV balloon dilatation using a CRISTAL, BALT balloon 22 mm over a 0.035-inch/260 extra-stiff Confida™ guidewire. Then, transseptal TMViV of an Edwards SAPIEN-3 23 mm valve with post-TMViV distal valve flaring, followed by transseptal implantation of a wireless pacemaker as the patient developed CHB. (d) TTE: both SAPIEN-3 valves are in mitral and aortic positions with normal flow across both valves. AF: atrial fibrillation, AR: aortic regurgitation, AS: aortic stenosis, AV: aortic valve, CHB: complete heart block, 3D: three-dimensional, MR: mitral regurgitation, MS: mitral stenosis, MV: mitral valve, MVA: mitral valve area, Pg: pressure gradient, TAVI: transcatheter aortic valve implantation, TMViV: transcatheter mitral valve-in-valve, TEE: transesophageal echocardiography, and TTE: transthoracic echocardiography.

**Figure 3:**
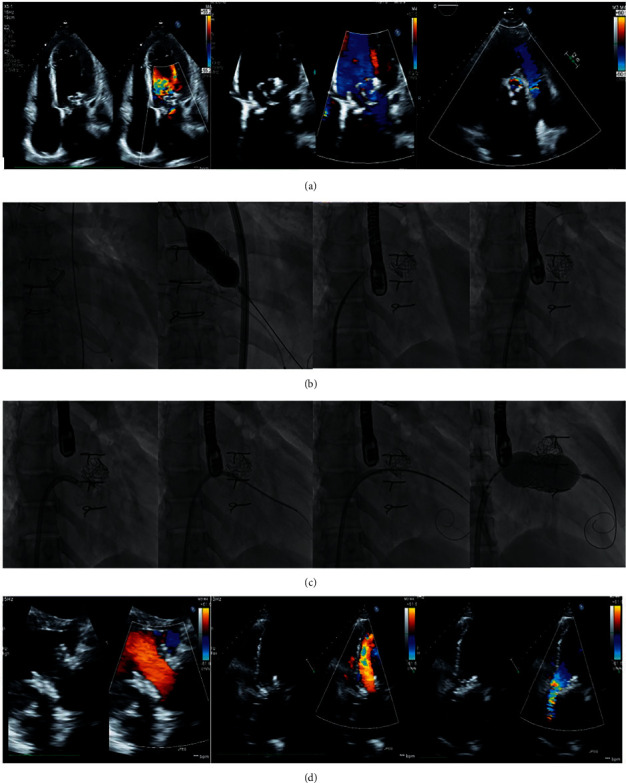
Transcatheter mitral valve in a degenerated EPIC™ (St. Jude Medical) valve (TMViV) with a concomitant transcatheter aortic valve implantation (TAVI). A 49-year-old old female patient with COPD had a degenerated bioprosthetic 29 mm EPIC™ MV and a degenerated bioprosthetic AV with an associated AF. TTE shows a degenerated bioprosthetic 29 mm EPIC™ MV (severe MS) and a degenerated bioprosthetic AV (severe AS). (b) Fluoroscopy shows the crossing of degenerated bioprosthetic AV with a 5F Amplatz left catheter over a 0.035-inch curved Terumo guidewire, followed by transaortic TAVI of an Edwards SAPIEN 3-26 mm valve during rapid pacing. Transseptal puncture using a 0-1 BRK™ within an 8.5F-SL sheath, followed by balloon dilatation of the transseptal puncture using a 12 mm balloon. (c) Fluoroscopy shows a transseptal TMViV of an Edwards SAPIEN-3 29-mm valve. First, an 8.5F-agilis™ sheath was flexed and directed towards the degenerated bioprosthetic MV. Second, a 5F-MP catheter over a 0.035-inch curved Terumo guidewire crossed the MV to the LV. Third, the wire was exchanged with a 0.035-inch/260 extra-stiff Confida™ guidewire. And finally, THV implantation. TTE: both SAPIEN-3 valves are in mitral and aortic positions with normal flow across both valves. AF: atrial fibrillation, AS: aortic stenosis, AV: aortic valve, COPD: chronic obstructive pulmonary disease, MS: mitral stenosis, MV: mitral valve, MVA: mitral valve area, Pg: pressure gradient, TAVI: transcatheter aortic valve implantation, TMViV: transcatheter mitral valve-in-valve, TTE: transthoracic echocardiography, and THV: transcatheter heart valve.

**Figure 4:**
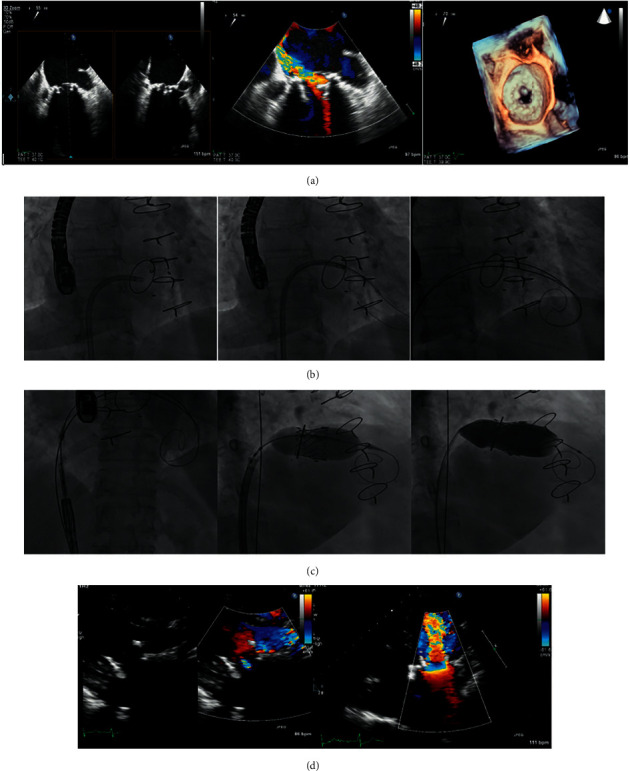
Transcatheter mitral valve in a degenerated Hancock™ II (Medtronic) valve (TMViV). A 66-year-old diabetic male patient, with ulcerative colitis, had a degenerated bioprosthetic 29 mm Hancock™ II MV with an associated AF. TEE and 3D-TEE show a degenerated bioprosthetic 29 mm Hancock™ II MV (severe MS and severe MR). (b) Fluoroscopy shows an 8.5F-agilis™ sheath was flexed and directed towards the degenerated bioprosthetic MV. A 5F-MP catheter over a 0.035-inch curved Terumo guidewire crossed the MV to the LV. The wire was exchanged with a 0.035-inch/260 extra-stiff Confida™ guidewire and a 0.035-inch/260 J-curve super-stiff Amplatz™ guidewire. (c) Fluoroscopy shows TMViV implantation of an Edwards SAPIEN-3 26 mm valve within the degenerated bioprosthetic MV, followed by post-TMViV distal valve flaring. D: TTE: an Edwards SAPIEN 3 26 mm valve in the MV position with normal Pg, and trivial with a valvular leak. AF: atrial fibrillation, 3D: three-dimensional, MR: mitral regurge, MS: mitral stenosis, MV: mitral valve, MVA: mitral valve area, Pg: pressure gradient, TMViV: transcatheter mitral valve-in-valve, TEE: transesophageal echocardiography, TTE: transthoracic echocardiography.

**Figure 5:**
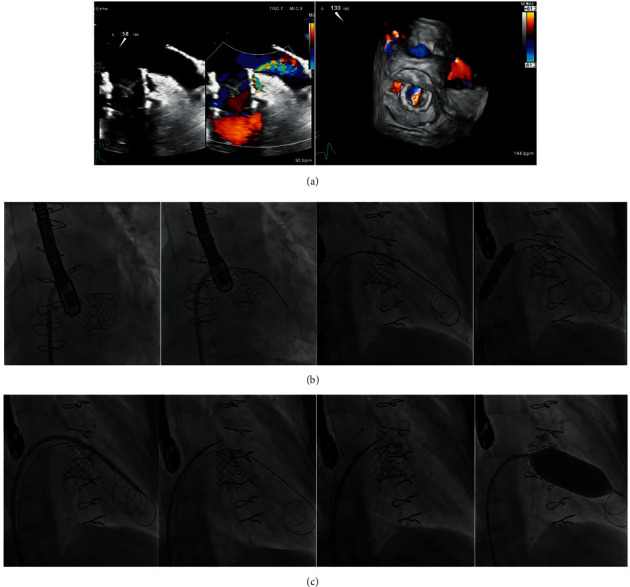
Transcatheter mitral valve in a St. Jude medical ring (TMViR) with a paravalvular ring leak. A 62-year-old female patient with a degenerated 27-mm MV St. Jude medical band post-MV repair and CABG with an associated AF. (a) TEE and color 3D-TEE 3 months post-TMViR with a SAPIEN-3 26 mm valve with a moderate to severe lateral paravalvular leak at 9–10 o'clock. (b) Fluoroscopy shows transseptal puncture through an 8.5F-SL sheath, followed by the crossing of a 5F-MP catheter on a 0.035-inch curved Terumo guidewire through the paravalvular leak from the LA side to the LV side. The guidewire was exchanged for two 0.035-inch/260 extra-stiff Confida™ guidewires, with balloon dilatation of the transseptal puncture using a 12 mm balloon. (c) Fluoroscopy shows the deployment of an Amplatzer™ muscular VSD 12 mm device and an Amplatzer™ vascular plug II 10 mm device, with post-TMViR balloon dilatation with an Edwards balloon 25 mm. The valve migrated and finally, the patient underwent surgical MV replacement. AF: atrial fibrillation; 3D: three-dimensional; LA: left atrium; LV: left ventricle; TMViR: transcatheter mitral valve-in-ring; TEE: transesophageal echocardiography; VSD: ventricular septal defect.

Among the 35 (55%) TMViV patients, 21 (60.0%) patients had a Carpentier–Edwards (Edwards Lifesciences) bioprosthesis (Figure 1), 7 (20.0%) patients had Mosaic® ULTRA™ (Medtronic) bioprosthesis (Figure 2), 5 (14.3%) patients had Epic™ (St Jude Medical) bioprosthesis (Figure 3), and 2 (5.7%) patients had Hancock™ II (Medtronic) bioprosthesis (Figure 4). Among the 16 (25.0%) TMViR patients, 6 (37.5%) patients had an Edwards Lifesciences ring, 4 (25.0%) patients had Medtronic ring, and 4 (25.0%) patients had St Jude Medical ring (Figure 5).

General anesthesia was used in 46 (71.9%) patients and conscious sedation was used in 18 (28.1%) patients. Pre-TMVR balloon dilatation was required in 10 (15.6%) patients [7 (53.9%) TMViMAC patients (*P* < 0.001)]. The large 29 mm SAPIEN-3 valve was more frequently used in TMViV and TMViMAC, and the smallest 23 mm SAPIEN-3 valve was more commonly used in TMViR (*P*=0.003^*∗*^). The procedure was elective in 54 (84.4%) patients, urgent in 6 (9.4%) patients, and emergency/salvage in 4 (6.3%) patients with a required cardiopulmonary bypass (CBP) in 6 (9.4%) patients. The mean procedural and fluoroscopy times were 58.7 ± 8.9 minutes and 41.1 ± 8.2 minutes, respectively, with no significant differences between the 3 groups (*P* > 0.05).

In the same set of TMVR implantation, 9 (14.1%) patients required concomitant PCI and 9 (14.1%) patients underwent concomitant planned TAVI, which were performed immediately before the TMVR procedure. TAVI was implanted retrograde transaortic in all patients except in 2 (5.7%) TMViV patients with inadequate peripheral circulation, for whom the transcaval approach was used. None of the patients required concomitant transcatheter tricuspid valve replacement (TTVR) or paravalvular leak closure in the same set of TMVR.

Technical success was reported in 62 (96.9%) patients;1 (6.3%) TMViR patient experienced valve embolization and underwent emergency/salvage cardiac surgery, and 1 (7.7%) TMViMAC patient experienced slight valve malposition without the need for second valve implantation or open-heart surgery. Most of the patients passed without procedural complications except the following: 7 (10.9%) patients showed access site hematoma (managed conservatively), 6 (9.4%) patients showed significant blood loss (required blood transfusion), and 3 (4.7%) patients showed complete heart block (CHB) (required temporary pacemakers).

During the in-hospital stay, 51 (79.7%) patients were successfully extubated on postoperative day 0 with a mean extubation time of 0.5 ± 0.2 days, a mean intensive care unit stay of 1.3 ± 0.5 days, and a mean total in-hospital stay of 3.2 ± 1.3 days. Seven (10.9%) patients showed pleural effusion, 2 (3.1%) patients experienced pneumonia, 1 (1.6%) TMViMAC patient developed stroke, and 4 (6.3%) patients required permanent pacing for CHB. No patient required reintervention, surgery, or exhibited mortality.

By the end of the study, the survival and follow-up data were available for all patients. At 3-month follow-up, 3 (4.7%) patients were rehospitalized for HF, 2 (3.1%) patients [1 (6.3%) TMViR patient and 1 (7.7%) TMViMAC patient] showed valve thrombosis and were treated with anticoagulation for 6 months, and 1 (6.3%) TMViR patient showed a significant paravalvular leak and underwent a trial for leak closure with devices with valve ballooning, followed by valve migration, and finally surgical MV replacement. At 6-month follow-up, 3 (4.7%) patients [1 (6.3%) TMViR patients and 2 (15.4%) TMViMAC patients] were rehospitalized for HF due to valve degeneration and required surgical MV replacement.

Thirty-five (54.7%) patients required a lifelong oral anticoagulant plus 3–6 months of an antiplatelet, and 29 (45.3%) patients were discharged on 1-year DAPT. Preprocedural, procedural, and postprocedural safety and quality measures for the studied patients are summarized in Table 2.

### 3.3. Echocardiographic Assessments of Valvular Function throughout Follow-Up

The postprocedural mean MV gradients were similar in all groups, with a significant reduction from 14.3 ± 5.3 mmHg preprocedure to 4.4 ± 1.2 mm Hg immediately postprocedure (*P* < 0.001), which remained unchanged throughout follow-up. Postprocedural MR was reduced significantly in all groups; TMViR patients were more likely to have residual nonsignificant MR. The LVEF and estimated systolic pulmonary artery pressure were improved significantly throughout follow-up in TMViV and TMViR patients (*P* < 0.05). LVOT-Pg did not change significantly in TMViV and TMViR patients (*P* > 0.05), while it increased significantly in TMViMAC patients (*P* < 0.001), without any need for alcohol septal ablation. Mostly, the iatrogenic atrial septal defect showed a spontaneous decrease in its size days after the procedure. Echocardiographic assessments of valvular function throughout the follow-up are summarized in Table 3.

## 4. Discussion

In the current study, TMVR was feasible in 64 high-surgical risk patients, with a technical success rate of 98.4% and without any recorded mortality. TMVR using the THV in the mitral position has been first reported in 2010 by Webb et al. [16] as a transseptal TMViV procedure. After that, TMVR was performed in 2012 using the CardiAQ valve (Edwards Lifesciences) [17]. In this cohort, the majority of treated patients were females, which was not unusual and was similar to other reports with the included percentages of women ranging from 59% to 77% [7, 8, 15, 18–21].

### 4.1. Challenges for TMVR

#### 4.1.1. Proper Patient Selection

TMVR-appropriate patient selection is challenging, with limited data regarding the inclusion criteria, exclusion criteria, and screen failure rates. In the Tendyne global feasibility study (Abbott Structural Heart) [22], only 100/332 patients were included, and in the Global Pilot study (Medtronic) [23], only 50/66 patients were selected, with a high screen failure rate. The most common exclusion criteria were large annulus, severe annular or leaflet calcification, high risk of LVOT obstruction, severe LV dysfunction, and intracardiac thrombus [22, 23]. In the current study, TMVR was limited to patients with high or prohibitive surgical risk with a mean surgical STS score of 9.2 ± 3.7% and without any recorded mortality. In one surgical study, mortality was observed in a similar STS database which found a 30-day mortality of 11.1% in 1096 patients who underwent redo MV surgery versus 6.5% mortality in 10145 patients who underwent first-time MV surgery (*P* < 0.0001) [24]. Nevertheless, no randomized clinical trials comparing TMVR with MV surgery outcomes are available.

#### 4.1.2. Optimal Sizing of the Transcatheter Heart Valve (THV)

In the current study, in addition to the valve size assessment by TEE and the Valve in Valve (Mitral) app, the cardiac CT-guided sizing of the THV was achieved in most patients. Naoum et al. addressed the implication of imaging techniques such as cardiac CT for the evaluation of patient eligibility, anatomical issues, and TMVR feasibility [25].

#### 4.1.3. Assessment of the Mitral Annular Calcification (MAC)

Despite performing 3D-CT evaluation for the current TMViMAC patients to confirm a continuous calcification of >50% of the MV circumference, 1 (7.7%) TMViMAC patient experienced slight valve malposition without the need for second valve implantation or open-heart surgery. The absence of a solid anatomic structure to anchor the THV in the mitral annulus represents a challenge for TMViMAC procedures, with an increased risk of valve malposition, migration, and/or embolization.

#### 4.1.4. Transseptal versus Transapical Access

In the Tendyne Global Feasibility and the Global Pilot studies [22, 23], the principal limitation was the transapical delivery, with a high 30-day mortality of 1/30 patients in the Tendyne study and 7/50 patients in the Intrepid study. In a meta-analysis on TMViV, the transapical approach was used in 55% of the patients with 5.7% in-hospital mortality and 23.4% 6-month mortality [26]. Also, in the Society of Thoracic Surgeons/American College of Cardiology/Transcatheter Valve Therapy (STS/ACC/TVT) Registry, the transapical approach was used in 44.8% of TMVR patients with in-hospital cardiac arrest of 4.7% [21]. Experiences with TAVI showed that the transapical approach was associated with higher bleeding risk and residual LV apex dysfunction [27, 28]. In all the current patients, the SAPIEN-3 valve was transeptally implanted to overcome apical access complications with a technical success rate of 98.4%. Webb et al. [29], who published their experience with the transeptally implanted SAPIEN-M3 THV (Edwards Lifesciences) in 10 patients with a technical success rate of 90%, were in agreement. Also, other numerous transapical and transseptal TMVR feasibility and safety single-arm studies are underway (TIARA-I, NCT02276547; High Life, NCT02974881; RELIEF, NCT02722551).

#### 4.1.5. Left Ventricular Outflow Tract (LVOT) Obstruction

At our institution, although LVOT-Pg was increased in TMViMAC patients, no LVOT obstruction required alcohol septal ablation at any time during follow-up. This could be explained by the 3D reconstructions of the TEE and cardiac CT, with the proper positioning of the THV with its outer skirt exactly into the plane of the bioprosthesis/ring/calcified annulus. In some studies, cardiac CT was vital in determining anatomical issues and in measuring the expected neo-LVOT area to assess the risk of TMVR-induced LVOT obstruction [25, 30, 31]. Several strategies to prevent or treat LVOT obstruction caused by TMVR have been described. The MITRAL trial [12, 13] has evaluated the role of preventive alcohol septal ablation in patients at risk for TMVR-induced LVOT obstruction. The LAMPOON trial (laceration of the anterior mitral leaflet to prevent outflow obstruction during TMVR) [32] evaluates the role of percutaneous laceration of the anterior leaflet to decrease the risk of TMVR-induced LVOT obstruction in TMViR and TMViMAC procedures. The SITRAL trial (surgical implantation of transcatheter valves) [33] assesses the role of the transatrial surgical access for TMVR in severe MAC. In a study by Yoon et al. [34], patients with LVOT obstruction had a higher mortality rate than patients without LVOT obstruction (34.6% vs 2.4%; *P* < 0.001). Various factors that contributed to LVOT obstruction include valve protrusion into the LV, anterior leaflet displacement, and a narrow aorto-mitral angle [35, 36].

#### 4.1.6. Risk of Valve Thrombosis and Early Degeneration

In the current cohort, the mean MV-Pg did not increase significantly during follow-up, opposing to the STS/ACC/TVT registry [21] that showed an increased transmitral gradient 30-day postprocedure. Whether this represented a true higher gradient from an early valve deterioration, an intraprocedural lower gradient from anesthesia, higher cardiac output from reduced MR, or a different assessment method (periprocedure catheterization/TEE versus follow-up TTE) was not known [21].

At the 3-month follow-up, valve thrombosis was reported in 2 (3.1%) TMViR patients and treated with 6-month anticoagulation. At the 6-month follow-up 3 (4.7%), patients showed valve degeneration and required surgical MV replacement. In the STS/ACC/TVT registry [21], the THV thrombosis was studied only at 30 days and was very low to be reported in 1/903 (0.02%) TMViV patient. After TMVR, the ideal anticoagulation duration was not known, and most trials recommend a minimum of 3–6 months of warfarin anticoagulation mimicking the recommendations for surgical bioprosthetic MV replacement [21]. Until further data are available, we recommended DAPT for one year in patients who did not require lifelong anticoagulation and a lifelong oral anticoagulant plus 3–6 months of an antiplatelet in patients who require lifelong anticoagulation. However, the risk and benefit ratio should be individualized.

### 4.2. Comparison between the Different Transcatheter Mitral Valve Replacement (TMVR) Types

#### 4.2.1. Transcatheter Mitral Valve-in-Valve (TMViV)

In this cohort, salvage TMViV with CBP was required only in 1 (2.9%) patient. This group had the highest procedural success and the lowest procedural complication rates as compared with TMViR and TMViMAC groups. Postprocedure MV function was excellent throughout the 6-month follow-up. By the end of the study, this group of patients reported no LVOT obstruction or mortality. The high procedural and clinical success rates were similar to the previous TMViV registries that showed an 82–100% procedural success rate and a 92%–95% clinical success rate [7–10, 21, 37]. The long-term outcomes in TMViV were good in some published series [19, 37], however, high mortality was recorded in the annual transcatheter valve therapy registry report of The Society of Thoracic Surgeons/American College of Cardiology [38] and some other reports [8, 15].

#### 4.2.2. Transcatheter Mitral Valve-in-Ring (TMViR)

In the current study, salvage TMViR was reported in 1 (6.3%) patient, and CBP was required in 2 (12.5%) patients. This group showed the lowest LVEF with an inferior overall outcome to TMViV but still superior to TMViMAC, and with a low procedural complication rate. At the 3-month follow-up, 2 (12.5%) patients were rehospitalized for HF; 1 (6.3%) patient showed valve thrombosis (treated with anticoagulation therapy) and 1 (6.3%) patient developed a paravalvular leak (required surgical MV replacement). In the last patient, although pre-TMViR balloon dilatation was achieved, the MV ring was not perfectly implanted between the commissures, resulting in a lack of proper valve anchoring. At the 6-month follow-up, 1 (6.3%) patient experienced early valve degeneration (required surgical MV replacement). Postprocedure MV function was excellent and was accepted till the 6-month follow-up. No mortality was recorded in this group till the end of the study. Procedural and valve success rates were similar to those in previously published reports [11, 21].

Similar to the TMViV group, this group of patients did not display any LVOT obstruction, opposing the STS/ACC/TVT registry [21] which suggested a higher risk of LVOT obstruction than TMViV, and explained this by the persistence of the native anterior mitral leaflet that was displaced into the LVOT.

In general, TMViR was more complex than TMViV due to the different types and shapes of the rings (rigid versus nonrigid, complete versus incomplete), which were usually not round and were predisposed to the paravalvular MR. We cannot compare the outcomes regarding the types of rings because of the small sample size and the nonvalidated methods to measure the MV area after the TMVR procedure. Previous reports stated that the THV in rigid and complete rings could result in valve under-expansion with a subsequent paravalvular leak. Similarly, the THV in bands/incomplete rings could result in valve embolization or a paravalvular leak [9, 10, 21]. More data are needed regarding the best method for accurate sizing of the mitral annulus and exact prediction of ring adoption of a circular valve. The optimal degree of oversizing for TMViR to avoid a paravalvular leak remains to be clarified.

#### 4.2.3. Transcatheter Mitral Valve-in-Mitral Annular Calcification (TMViMAC)

In this cohort, salvage TMViMAC was reported in 2 (15.4%) patients, and CBP was required in 3 (23.1%) patients. This group had the lowest technical, procedural, and valve success rates, as well as the highest in-hospital, 3-month follow-up, and 6-month follow-up complications. Periprocedural, 1 (7.7%) patient developed slight valve malposition; at 3-month follow-up, 1 (7.7%) patient experienced valve thrombosis (treated with anticoagulation therapy);at 6-month follow-up, 2 (15.4%) patients experienced early valve degeneration (required surgical MV replacement). Postprocedure MV function was excellent but showed a nonsignificant increase of the transmitral gradient at 6-month follow-up. No mortality was recorded in this group till the end of the study.

The evidence from prior registries displayed the lowest procedural and valve success rates in TMViMAC as compared to TMViV and TMViR patients [9–11, 18, 19, 21, 39]. The reasons included several comorbidities and technical challenges. Among the technical challenges are the difficult positioning of the circular THV into the saddle oval-shaped MV annulus with the subsequent paravalvular leak, the deficient calcified annular area with possible embolization, and the presence of the subvalvular apparatus with probable LVOT interaction.

By the end of this study, the TMViMAC patients showed a significant increase in LVOT-Pg, but still less than that observed by Yoon et al. [11] and by the Global MAC Registry [18, 39]. In these 2 registries, the LVOT obstruction was the strongest predictor of 30-day and 1-year mortality [18, 39]. In TMViMAC, the best method for mitral annulus sizing, the calcium burden needed, and the optimal height of implantation concerning the mitral annular plane are still to be clarified. Presently, TMViMAC should be performed only in patients without surgical treatment options.

### 4.3. Mortality

In this cohort, fortunately, no mortality was recorded till the end of the study. In the STS/ACC/TVT Registry [21], although the in-hospital mortality was 4.8%, it remained with a nonsignificant value (*P* > 0.05) (supplementary table (available here)). In the current study, the lower mortality rate compared with the STS/ACC/TVT Registry could be related to younger age, less STS risk score, improved experience in patients selection, fewer MS patients, largest MV area, proper sizing techniques including 3D-CT to decrease the risk of LVOT obstruction, less urgent/salvage patients, application of the transseptal approach, usage of the last version SAPIEN-3 valve with balloon flaring of its distal end, and early reintervention with any valve complications (Supplementary table).

### 4.4. Study Strengths and Limitations

#### 4.4.1. Strength

The approach was transseptal in all patients, and neither contrast nor pacing was used for the TMVR procedure. Clinical follow-up was 100% complete at 3 months and 6 months and included all information on survival, echocardiographic analyses, rehospitalization, the need for MV re-intervention/surgery, and mortality.

#### 4.4.2. Limitations

This is a single-center experience, and a larger population with longer follow-up would be necessary to assess the durability of these valves. Still there is a lack of applicability to other patients with low, intermediate, or high (but operable) surgical risk.

## 5. Conclusions

Transseptal TMVR is a feasible and safe approach in patients with high surgical risk, with a reasonable short- and mid-term efficacy. In patients with high surgical risk, TMViV and TMViR are the first-line approaches in the treatment of failing mitral bioprosthesis or annuloplasty rings. However, TMViMAC is still associated with a higher complication rate, and the outcome seems encouraging in carefully selected patients.

## Figures and Tables

**Table 1:** Baseline demographic, clinical, and echocardiographic characteristics of the studied patients.

Preprocedural characteristics	TMVR total *N*: 64	Groups			*P* value
		TMViV *N*: 35 (55%)	TMViR *N*: 16 (25%)	TMViMAC *N*: 13 (20%)	
*Demographic characteristics*					
Age (years)	62.7 ± 16.1	58.0 ± 19.5	66.2 ± 7.7	71.1 ± 7.7	0.024^*∗*^
Female gender	35 (54.7%)	22 (62.9%)	8 (50.0%)	5 (38.5%)	0.291
Weight (kg)	61.7 ± 10.5	61.6 ± 11.0	64.3 ± 11.2	59.0 ± 8.0	0.404
Height (m)	1.6 ± 0.15	1.6 ± 0.2	1.6 ± 0.1	1.6 ± 0.1	0.316
BSA (m^2^)	3.9 ± 18.5	1.6 ± 0.2	1.6 ± 0.1	1.5 ± 0.1	0.087
Risk factors (high risk)					
Diabetes mellitus	41 (64.1%)	17 (48.6%)	14 (87.5%)	10 (76.9%)	0.055
Hypertension	30 (46.9%)	13 (37.1%)	8 (50.0%)	9 (69.2%)	0.135
COPD/home oxygen	13 (20.3%)	4 (11.4%)	7 (43.8%)	2 (15.4%)	0.026^*∗*^
CKD/renal transplant	20 (31.3%)	3 (8.6%)	7 (43.8%)	10 (76.9%)	<0.001^*∗*^
Decompensating liver	4 (6.3%)	1 (2.9%)	2 (12.5%)	1 (7.7%)	0.406
Stroke	25 (39.1%)	8 (22.9%)	9 (56.3%)	8 (61.5%)	0.014^*∗*^
Peripheral vascular disease	7 (10.9%)	2 (5.7%)	3 (18.8%)	2 (15.4%)	0.325
Chronic anemia	9 (14.1%)	2 (5.7%)	1 (6.3%)	6 (46.2%)	<0.001^*∗*^
Previous cardiac history					
Arrythmias (SVT, AF, VT, CHB)	35 (54.7%)	19 (54.3%)	7 (43.8%)	9 (69.2%)	0.070
Prior myocardial infarction	19 (29.7%)	0 (0.0%)	12 (75.0%)	7 (53.9%)	<0.001^*∗*^
Prior PCI	9 (14.1%)	1 (2.9%)	3 (18.8%)	5 (38.5%)	0.006^*∗*^
Prior CABG	18 (28.1%)	1 (2.9%)	12 (75.0%)	5 (38.5%)	<0.001^*∗*^
Prior AV replacement	9 (14.1%)	6 (17.1%)	0 (0.0%)	3 (23.1%)	0.047^*∗*^
Prior congenital surgery	1 (1.6%)	1 (2.9%)	0 (0.0%)	0 (0.0%)	0.656
Prior PPM/ICD	10 (15.6%)	7 (20.0%)	0 (0.0%)	3 (23.1%)	0.134
Previous anticoagulation	23 (35.9%)	8 (22.9%)	8 (50.0%)	7 (53.9%)	0.055
Prior HF hospitalization in last 12 months	42 (65.6%)	23 (65.7%)	13 (81.3%)	6 (46.2%)	0.141
Porcelain aorta	3 (4.7%)	0 (0.0%)	0 (0.0%)	3 (23.1%)	0.002^*∗*^
Timing of last MV surgery (Ys)	7.6 ± 5.5	9.9 ± 4.6	8.6 ± 4.0	0.0 ± 0.0	
STS score (%)	9.2 ± 3.7	8.6 ± 4.1	9.6 ± 3.3	10.8 ± 5.6	0.442

*Clinical presentations*					
Presentation					
Palpitation	12 (18.8%)	5 (14.3%)	3 (18.8%)	4 (30.8%)	0.429
Dyspnea	64 (100.0%)	35 (100.0%)	16 (100.0%)	13 (100.0%)	0.656
Chest pain	2 (3.1%)	2 (5.7%)	0 (0.0%)	0 (0.0%)	0.425
Stroke	1 (1.6%)	1 (2.9%)	0 (0.0%)	0 (0.0%)	0.656
NYHA-FC					
III	17 (26.6%)	9 (25.7%)	4 (25.0%)	4 (30.8%)	0.939
IV	47 (73.4%)	25 (71.4%)	13 (81.3%)	9 (69.2%)	

*Echocardiography data*					
Pathology of MV lesions					
Degeneration	51 (79.7%)	35 (100.0%)	16 (100.0%)	0 (0.0%)	<0.001^*∗*^
Calcification	13 (20.3%)	0 (0.0%)	0 (0.0%)	13 (100.0%)	<0.001^*∗*^
Previous infective endocarditis	7 (10.9%)	5 (14.3%)	2 (12.5%)	0 (0.0%)	0.361
MV lesion					
MS	20 (31.3%)	14 (40.0%)	4 (25.0%)	2 (15.4%)	0.043^*∗*^
MR	6 (9.4%)	1 (2.9%)	5 (31.3%)	0 (0.0%)	<0.001^*∗*^
Both	38 (59.4%)	20 (57.1%)	7 (43.8%)	11 (84.6%)	0.003^*∗*^
Severity of MS					
Mild	2 (3.1%)	0 (0.0%)	2 (12.5%)	0 (0.0%)	<0.001^*∗*^
Moderate	6 (9.4%)	0 (0.0%)	3 (18.8%)	3 (23.1%)	
Severe	50 (78.1%)	34 (97.1%)	6 (37.5%)	10 (76.9%)	
MV area (cm^2^)	1.5 ± 0.6	1.1 ± 0.3	2.1 ± 0.8	1.6 ± 0.5	<0.001^*∗*^
Mean MV-Pg (mmHg)	14.3 ± 5.3	14.8 ± 5.1	12.1 ± 4.6	15.5 ± 6.4	0.162
Severity of MR					
Trivial/Mild	3 (4.7%)	3 (8.6%)	0 (0.0%)	0 (0.0%)	0.319
Moderate	15 (23.4%)	8 (22.9%)	5 (31.3%)	2 (15.4%)	
Severe	26 (40.6%)	10 (28.6%)	7 (43.8%)	9 (69.2%)	
Type of MR					
Paravalvular	3 (4.7%)	0 (0.0%)	3 (18.8%)	0 (0.0%)	0.022^*∗*^
Valvular	41 (64.1%)	21 (60.0%)	9 (56.3%)	11 (84.6%)	

AF: atrial fibrillation, AV: aortic valve, BSA: body surface area, CABG: coronary artery bypass graft, CHB: complete heart block, CKD: chronic kidney disease, COPD: chronic obstructive pulmonary disease, HF: heart failure, MR: mitral regurgitation, MS: mitral stenosis, MV: mitral valve, NYHA-FC: New York heart association functional class, PCI: percutaneous coronary intervention, Pg: pressure gradient, PPM/ICD: permanent pacemaker/implantable cardioverter-defibrillator, STS: Society of Thoracic Surgeons, SVT: supraventricular tachycardia, TMVR: transcatheter mitral valve replacement, TMViV: transcatheter mitral valve-in-valve, TMViR: transcatheter mitral valve-in-ring, TMViMAC: transcatheter mitral valve-in-mitral annular calcification, VT: ventricular tachycardia.

**Table 2:** Preprocedural, procedural, and postprocedural safety and quality measures for the studied patients.

Procedural characteristics	TMVR total *N*: 64	Groups			*P* value
		TMViV *N*: 35 (55%)	TMViR *N*: 16 (25%)	TMViMAC *N*: 13 (20%)	
*Pre-procedural data for preparation*					
Prior MV bioprosthesis types					
Carpentier–Edwards (Edwards Lifesciences)		21 (60.0%)			
Mosaic® ULTRA™ (Medtronic)		7 (20.0%)			
Epic™ (St. Jude medical)		5 (14.3%)			
Hancock™ II (Medtronic)		2 (5.7%)			
Prior MV ring types					
Edwards Lifesciences			6 (37.5%)		
Medtronic			4 (25.0%)		
St Jude Medical			4 (25.0%)		

*Procedural data*					
Anesthesia					
General anesthesia	46 (71.9%)	23 (65.7%)	12 (75.0%)	11 (84.6%)	0.411
Conscious sedation	18 (28.1%)	12 (34.3%)	4 (25.0%)	2 (15.4%)	
Pre-TMVR balloon dilatation	10 (15.6%)	1 (2.9%)	2 (12.5%)	7 (53.9%)	<0.001^*∗*^
Pre-TMVR balloon size (mm)	25.6 ± 2.4	22.0 ± 0.0	23.5 ± 2.1	26.7 ± 1.6	0.039^*∗*^
Post-TMVR distal valve flaring	64 (100.0%)	35 (100.0%)	16 (100.0%)	13 (100.0%)	
Valve type (Edward SAPIEN-3)	64 (100.0%)	35 (100.0%)	16 (100.0%)	13 (100.0%)	
Valve size (mm)					0.003^*∗*^
23	7 (10.9%)	2 (5.7%)	5 (31.3%)	0 (0.0%)	
26	33 (51.6%)	18 (51.4%)	9 (56.3%)	6 (46.2%)	
29	24 (37.5%)	15 (42.9%)	2 (12.5%)	7 (53.9%)	
Concomitant procedures					
Concomitant PCI	9 (14.1%)	3 (8.6%)	2 (12.5%)	4 (30.8%)	0.142
Concomitant TAVI	9 (14.1%)	7 (20.0%)	0 (0.0%)	2 (15.4%)	0.161
Procedure status					
Elective	54 (84.4%)	32 (91.4%)	13 (81.3%)	9 (69.2%)	0.071
Urgent	6 (9.4%)	2 (5.7%)	2 (12.5%)	2 (15.4%)	
Emergency/salvage	4 (6.3%)	1 (2.9%)	1 (6.3%)	2 (15.4%)	
Mechanical assist devices (CBP)	6 (9.4%)	1 (2.9%)	2 (12.5%)	3 (23.1%)	0.153
Procedural time (minutes)	58.7 ± 8.9	58.9 ± 9.2	58.1 ± 7.0	59.2 ± 10.5	0.941
Fluoroscopy time (minutes)	41.1 ± 8.2	41.1 ± 8.9	40.6 ± 6.6	41.7 ± 8.4	0.940
Technical success	62 (96.9%)	35 (100.0%)	15 (93.8%)	12 (92.3)	0.711
Procedural complications					
Access site complications	7 (10.9%)	2 (5.7%)	2 (12.5%)	3 (23.1%)	0.225
Significant blood loss/blood transfusion	6 (9.4%)	2 (5.7%)	2 (12.5%)	2 (15.4%)	0.525
CHB/new pacemaker	3 (4.7%)	1 (2.9%)	0 (0.0%)	2 (15.4%)	0.017^*∗*^
Valve malposition/embolization/thrombosis	2 (3.1%)	0 (0.0%)	1 (6.3%)	1 (7.7%)	0.091
MV reintervention/surgery	1 (1.6%)	0 (0.0%)	0 (0.0%)	0 (0.0%)	0.136
Procedural mortality	0 (0.0%)	0 (0.0%)	0 (0.0%)	0 (0.0%)	–

*In-hospital outcome*					
Timing of extubation (days)	0.5 ± 0.2	0.4 ± 0.1	0.5 ± 0.3	0.5 ± 0.5	0.056
ICU stay (days)	1.3 ± 0.5	1.3 ± 0.6	1.3 ± 0.5	1.5 ± 0.5	0.273
Total in-hospital stay (days)	3.2 ± 1.3	3.3 ± 1.4	2.9 ± 1.1	3.6 ± 1.5	0.324
In-hospital complications					
Pleural effusion	7 (10.9%)	3 (8.6%)	2 (12.5%)	2 (15.4%)	0.225
CHB/PPM	4 (6.3%)	2 (5.7%)	1 (6.3%)	1 (7.7%)	0.525
Pneumonia	2 (3.1%)	2 (5.7%)	0 (0.0%)	0 (0.0%)	0.425
Stroke	1 (1.7%)	0 (0.0%)	0 (0.0%)	1 (7.7%)	0.136
Valve malposition/migration/embolization/thrombosis	0 (0.0%)	0 (0.0%)	0 (0.0%)	0 (0.0%)	–
MV reintervention/surgery	0 (0.0%)	0 (0.0%)	0 (0.0%)	0 (0.0%)	–
Mortality	0 (0.0%)	0 (0.0%)	0 (0.0%)	0 (0.0%)	–

*3-month complications and outcome*					
Valve thrombosis	2 (3.1%)	0 (0.0%)	1 (6.3%)	1 (7.7%)	0.185
Paravalvular leak	1 (1.6%)	0 (0.0%)	1 (6.3%)	0 (0.0%)	0.277
Hospitalization for HF	3 (4.7%)	0 (0.0%)	2 (12.5%)	1 (7.7%)	0.043^*∗*^
MV reintervention/surgery	1 (1.6%)	0 (0.0%)	1 (6.3%)	0 (0.0%)	0.277
Mortality	0 (0.0%)	0 (0.0%)	0 (0.0%)	0 (0.0%)	–

*6-month complications and outcome*					
Valve degeneration	3 (4.7%)	0 (0.0%)	1 (6.3%)	2 (15.4%)	0.035^*∗*^
Hospitalization for HF	3 (4.7%)	0 (0.0%)	1 (6.3%)	2 (15.4%)	0.035^*∗*^
MV reintervention/surgery	3 (4.7%)	0 (0.0%)	1 (6.3%)	2 (15.4%)	0.035^*∗*^
Mortality	0 (0.0%)	0 (0.0%)	0 (0.0%)	0 (0.0%)	–

*Periprocedural antithrombotic therapy*					
DAPT (aspirin-clopidogrel or aspirin-ticagrelor)	29 (45.3%)	16 (45.7%)	9 (56.3%)	4 (30.8%)	0.115
Anticoagulant and antiplatelet	35 (54.7%)	19 (54.3%)	7 (43.8%)	9 (69.2%)	0.070

CBP: cardiopulmonary bypass, CHB: complete heart block, DAPT: dual antiplatelet therapy, HF: heart failure, ICU: intensive care unit, MV: mitral valve, PCI: percutaneous coronary intervention, PPM: permanent pacemaker, TAVI: transcatheter aortic valve implantation, TMVR: transcatheter mitral valve replacement, TMViV: transcatheter mitral valve-in-valve, TMViR: transcatheter mitral valve-in-ring, TMViMAC: transcatheter mitral valve-in-mitral annular calcification, TTVR: transcatheter tricuspid valve replacement.

**Table 3:** Echocardiographic assessments of valvular function throughout follow-up.

	TMVR total *N*: 64	Groups			*P*-value
		TMViV *N*: 35 (55%)	TMViR *N*: 16 (25%)	TMViMAC *N*: 13 (20%)	
*MV mean Pg (mmHg)*					
Preprocedure	14.3 ± 5.3	14.8 ± 5.1	12.1 ± 4.6	15.5 ± 6.4	0.162
Immediately/In-hospital	4.4 ± 1.2	4.2 ± 1.3	4.4 ± 1.0	4.6 ± 1.0	0.618
	<0.001^*∗*^	<0.001^*∗*^	<0.001^*∗*^	<0.001^*∗*^	
3-month postprocedure	4.7 ± 1.3	4.9 ± 1.4	4.6 ± 1.0	5.1 ± 1.4	0.554
	<0.001^*∗*^	<0.001^*∗*^	<0.001^*∗*^	<0.001^*∗*^	
6-month postprocedure	5.4 ± 1.6	4.9 ± 1.7	5.4 ± 1.5	5.5 ± 1.4	0.438
	<0.001^*∗*^	<0.001^*∗*^	<0.001^*∗*^	<0.001^*∗*^	

*MR*					
Preprocedure					0.319
(i) Trivial/Mild	3 (4.7%)	3 (8.6%)	0 (0.0%)	0 (0.0%)	
(ii) Moderate	15 (23.4%)	8 (22.7%)	5 (31.3%)	2 (15.4%)	
(iii) Sever	26 (40.6%)	10 (28.6%)	7 (43.8%)	9 (69.2%)	
Immediately/In-hospital (Trivial/Mild)	10 (15.6%)	3 (8.6%)	5 (31.3%)	2 (15.4%)	0.215
	<0.001^*∗*^	<0.001^*∗*^	<0.001^*∗*^	0.001^*∗*^	
3-month postprocedure (trivial/mild/moderate)	11 (17.2%)	3 (8.6%)	6 (37.5%)	2 (15.4%)	0.074
	<0.001^*∗*^	<0.001^*∗*^	<0.001^*∗*^	0.002^*∗*^	
6-month postprocedure (trivial/mild/moderate)	17 (26.6%)	9 (25.7%)	6 (37.5%)	2 (15.4%)	0.670
	<0.001^*∗*^	<0.001^*∗*^	<0.001^*∗*^	0.002^*∗*^	

*LVEF (%)*					
Preprocedure	48.8 ± 15.8	49.3 ± 13.8	34.4 ± 11.4	65.2 ± 5.8	<0.001^*∗*^
Immediately/In-hospital	50.7 ± 14.8	51.6 ± 12.9	36.8 ± 10.7	65.5 ± 5.9	<0.001^*∗*^
	<0.001^*∗*^	<0.001^*∗*^	0.006^*∗*^	0.610	
3-month postprocedure	52.3 ± 14.0	52.7 ± 12.2	40.3 ± 11.8	65.8 ± 62	<0.001^*∗*^
	<0.001^*∗*^	<0.001^*∗*^	<0.001^*∗*^	0.292	
6-month postprocedure	53.8 ± 13.0	54.6 ± 11.1	42.4 ± 11.9	65.9 ± 5.6	<0.001^*∗*^
	<0.001^*∗*^	<0.001^*∗*^	<0.001^*∗*^	0.157	

*ESPAP (mm Hg)*					
Pre-procedure	67.9 ± 18.8	69.7 ± 19.3	61.6 ± 20.4	70.8 ± 14.6	0.302
Immediately/In-hospital	61.0 ± 15.4	60.5 ± 14.3	56.6 ± 17.4	67.5 ± 14.3	0.05^*∗*^
	<0.001^*∗*^	<0.001^*∗*^	0.038^*∗*^	0.072	
3-month postprocedure	55.0 ± 15.1	52.6 ± 12.7	50.8 ± 16.7	66.5 ± 14.6	0.006^*∗*^
	<0.001^*∗*^	<0.001^*∗*^	0.002^*∗*^	0.063	
6-month postprocedure	51.7 ± 13.6	48.8 ± 9.8	47.7 ± 14.6	66.0 ± 14.7	0.001^*∗*^
	<0.001^*∗*^	<0.001^*∗*^	0.001^*∗*^	0.059	

*LVOT Pg (mmHg)*					
Pre-procedure	3.14 ± 1.8	2.7 ± 1.8	2.7 ± 1.3	4.9 ± 0.9	<0.001^*∗*^
Immediately/In-hospital	4.0 ± 1.8	3.0 ± 1.4	3.6 ± 1.5	6.1 ± 0.8	<0.001^*∗*^
	<0.001^*∗*^	0.086	0.59	<0.001^*∗*^	
3-month postprocedure	4.1 ± 1.9	3.1 ± 1.4	3.9 ± 1.3	6.45 ± 1.1	<0.001^*∗*^
	<0.001^*∗*^	0.055	0.057	<0.001^*∗*^	
6-month postprocedure	4.4 ± 2.2	3.1 ± 1.5	4.0 ± 1.3	7.5 ± 1.9	<0.001^*∗*^
	<0.001^*∗*^	0.061	0.053	<0.001^*∗*^	

ESPAP: estimated systolic pulmonary artery pressure, LVEF: left ventricular ejection fraction, LVOT: left ventricular outflow tract, MR: mitral regurgitation, MV: mitral valve, Pg: pressure gradient, TMVR: transcatheter mitral valve replacement, TMViV: transcatheter mitral valve-in-valve, TMViR: transcatheter mitral valve-in-ring, TMViMAC: transcatheter mitral valve-in-mitral annular calcification.

## Data Availability

All patients data are available.

## Abbreviations

3D: Three-dimensional
BRK™: Brockenbrough needle
CBP: Cardiopulmonary bypass
CHB: Complete heart block
CT: Computed tomography
DAPT: Dual antiplatelet therapy
FDA: Food and Drug administration
HF: Heart failure
LA: Left atrium
LV: Left ventricle
LVEF: Left ventricular ejection fraction
LVOT: Left ventricular outflow tract
MAC: Mitral annular calcification
MR: Mitral regurgitation
MV: Mitral valve
MS: Mitral stenosis
NYHA-FC: New York heart association-functional class
PCI: Primary coronary intervention
Pg: Pressure gradient
STS: Society of Thoracic Surgeons
TAVI: Transcatheter aortic valve implantation
TEE: Transesophageal echocardiography
THV: Transcatheter heart valve
TMVR: Transcatheter mitral valve replacement
TMViV: Transcatheter mitral valve-in-valve
TMViR: Transcatheter mitral valve-in-ring
TMViMAC: Transcatheter mitral valve-in-mitral annular calcification
TTE: Transthoracic echocardiography
TTVR: Transcatheter tricuspid valve replacement.

## Supplementary Materials

Supplementary Materials Supplementary table: comparison between this study and the Society of Thoracic Surgeons/American College of Cardiology/Transcatheter Valve Therapy (STS/ACC/TVT) registry.
