# Supplementary material for: Transcatheter Mitral Valve Replacement in High-Surgical Risk Patients: A Single-Center Experience and Outcome
Source: J Interv Cardiol. 2022 Jun 22;2022:6587036. doi: 10.1155/2022/6587036 (PMC9242753; doi:10.1155/2022/6587036)
Supplement: Supplementary Materials — Supplementary table: comparison between this study and the Society of Thoracic Surgeons/American College of Cardiology/Transcatheter Valve Therapy (STS/ACC/TVT) registry. [file 6587036.f1.docx]

**SUPPLEMENTARY TABLE:**

Supplementary table: Comparison between this study and the Society of Thoracic Surgeons/American College of Cardiology/Transcatheter Valve Therapy (STS/ACC/TVT) registry

| **Pre-procedural Characteristics** | **TMVR**  **Total (N: 64)** | **TMVR (STS/ACC/TVT**  **Registry)**  **Total (N: 903)** | **P-value** |
| --- | --- | --- | --- |
| **Demographic Characteristics** | | | |
| **Male Gender** | **29 (45.3%)** | **368 (40.7%)** | **0.559** |
| **Age (Ys)** | **62.7 ± 16.1** | **75 ± 10.4** | **<0.001*** |
| **Risk factors (High risk)** |  |  |  |
| **Diabetes Mellitus** | **41 (64.1%)** | **255 (28.3%)** | **<0.001*** |
| **COPD/Home oxygen** | **13 (20.3%)** | **153 (17%)** | **0.604** |
| **CKD/Renal transplant** | **20 (31.25%)** | **528 (58.9%)** | **<0.001*** |
| **Stroke** | **25 (39.1%)** | **195 (21.6%)** | **0.002*** |
| **Previous cardiac history** |  | |  |
| **Arrhythmias (SVT, AF, VT, CHB)** | **35 (54.7%)** | **587 (65%)** | **0.126** |
| **Prior CABG** | **18 (28.1%)** | **364 (40.8%)** | **0.073** |
| **Prior AV replacement** | **9 (14.06%)** | **194 (21.6%)** | **0.211** |
| **Prior PPM/ICD** | **10 (15.6%)** | **367 (40.7%)** | **<0.001*** |
| **Previous anticoagulation** | **23 (35.9%)** | **447 (51.5%)** | **0.049*** |
| **Porcelain aorta** | **3 (4.7%)** | **22 (2.4%)** | **0.491** |
| **STS -score (%)** | **8.4 ± 9.8** | **10 ± 6.5** | **0.068** |
| **Clinical Presentations** | | | |
| **NYHA-FC** |  | |  |
| **III** | **17 (26.6%)** | **493 (55.1%)** | **<0.001*** |
| **IV** | **47 (73.4%)** | **308 (34.5%)** |  |
| **Echocardiographic Data** | | | |
| **MS** | **20 (31.3%)** | **598 (67.6%)** | **<0.001*** |
| **MV area (cm^2^)** | **1.5 ± 0.6** | **1.3 ± 0.7** | **0.026*** |
| **MV mean Pg (mmHg)** | **14.3 ± 5.3** | **13.8 ± 5.2** | **0.066** |
| **MR** |  | |  |
| **Trivial/Mild** | **3 (4.7%)** | **247 (27.7%)** | **0.003*** |
| **Moderate** | **15 (23.4%)** | **220 (24.6%)** |  |
| **Severe** | **26 (40.6%)** | **342 (38.3%)** |  |
| **LVEF** | **48.8 ± 15.8** | **53.3 ± 8.4** | **0.057** |
| **ESPAP** | **67.9 ± 18.8** | **63.9 ± 13.2** | **0.054** |
| **Procedural Characteristics** | | | |
| **Procedure status** |  | |  |
| **Elective** | **54 (84.4%)** | **687 (76.2)** | **0.173** |
| **Urgent** | **6 (9.4%)** | **205 (22.7)** | **0.019*** |
| **Emergency/salvage** | **4 (6.3%)** | **10 (1.1)** | **0.005*** |
| **Mechanical assist devices** | **6 (9.4%)** | **46 (5.1%)** | **0.238** |
| **Access** |  | |  |
| **Transseptal** | **64 (100%)** | **389 (43.1%)** | **<0.001*** |
| **Transapical** | **0 (0%)** | **404 (****44.8%)** | **<0.001*** |
| **Others** | **0 (0%)** | **110 (12.1%)** | **0.006*** |
| **Pre-TMVR balloon dilatation** | **10 (15.6%)** | **312 (35.4%)** | **0.003*** |
| **Post-TMVR distal valve flaring** | **64 (100.0%)** | **201 (22.7%)** | **<0.001*** |
| **Valve type (Edward Sapiene 3)** |  | |  |
| **SAPIENE** | **0 (0.0%)** | **36 (4%)** | **0.198** |
| **SAPIENE XT** | **0 (0.0%)** | **364 (40.3%)** | **<0.001*** |
| **SAPIENE 3** | **64 (100.0%)** | **468 (51.8%)** | **<0.001*** |
| **Valve size (mm)** |  | |  |
| **23 mm** | **7 (10.9%)** | **90 (10.2%)** | **0.973** |
| **26 mm** | **33 (51.6%)** | **350 (39.8%)** | **0.059** |
| **29 mm** | **24 (37.5%)** | **439 (49.9%)** | **0.112** |
| **Procedural time (minutes)** | **58.7 ± 8.9** | **120 ± 68** | **<0.001*** |
| **Technical success** | **62 (96.9%)** | **849 (94.1%)** | **0.504** |
| **Procedural complications** |  | |  |
| **Access site complications** | **7 (10.9%)** | **30 (3.3%)** | **0.006*** |
| **Significant blood loss/blood transfusion** | **6 (9.4%)** | **89 (10%)** | **0.926** |
| **CHB/New pacemaker** | **3 (4.7%)** | **11 (1.2%)** | **0.088** |
| **Valve malposition/embolization/thrombosis** | **2 (3.13%)** | **11 (1.2%)** | **0.473** |
| **MV re-intervention/surgery** | **1 (1.56%)** | **25 (2.8%)** | **0.860** |
| **Cardiac perforation** | **0 (0.0%)** | **19 (2.1%)** | **0.480** |
| **Procedural Mortality** | **0 (0.0%)** | **0 (0.0%)** | **-** |
| **In-hospital outcome** | | | |
| **MV mean Pg (mmHg)** | **4.4 ± 1.2** | **4.0 ± 1.8** | **0.080** |
| **MR (Moderate)** |  |  |  |
| **Mild** | **10 (15.6%)** | **173 (19.3%)** | **0.595** |
| **Moderate** | **0 (0.0%)** | **41 (4.5%)** | **0.155** |
| **Sever** | **0 (0.0%)** | **9 (1%)** | **0.898** |
| **In-hospital complications** |  |  |  |
| **CHB/AF/PPM** | **4 (6.3%)** | **23 (2.5%)** | **0.179** |
| **Stroke** | **1 (1.6%)** | **17 (1.9%)** | **0.768** |
| **Valve malposition/migration/embolization/thrombosis** | **0 (0.0%)** | **0 (0.0%)** | **-** |
| **Valve endocarditis** | **0 (0.0%)** | **0 (0.0%)** | **-** |
| **MV re-intervention/surgery** | **0 (0.0%)** | **0 (0.0%)** | **-** |
| **Cardiac arrest** | **0 (0.0%)** | **43 (****4.8%)** | **0.141** |
| **Medications** |  |  |  |
| **Aspirin alone** | **0 (0.0%)** | **431 (53.3%)** | **<0.001*** |
| **DAPT (Aspirin-clopidogrel or aspirin-ticagrelor)** | **29 (45.3%)** | **235 (29.1%)** | **0.001*** |
| **Anticoagulants** | **35 (54.7%)** | **573 (70.9%)** | **0.204** |

*AF: Atrial fibrillation, CABG: Coronary artery bypass graft, CHB: Complete heart block, CKD: Chronic kidney disease, ESPAP: Estimated systolic pulmonary artery pressure, LVEF: Left ventricular ejection fraction, MR: Mitral regurgitation, MS: Mitral stenosis, MV: Mitral valve, NYHA-FC: New York Heart Association Functional Class, Pg: Pressure gradient, PPM/ICD: Permanent pacemaker/Implantable cardioverter-defibrillator, STS: Society of Thoracic Surgeons,* STS/ACC/TVTR: Society of Thoracic Surgeons/American College of Cardiology/Transcatheter Valve Therapy Registry, *SVT: Supraventricular tachycardia, TMVR: Transcatheter mitral valve replacement, VT: Ventricular tachycardia.*
